# Antenatal diagnosis of vasa previa: report of three cases in an African setting

**DOI:** 10.11604/pamj.2020.37.24.25663

**Published:** 2020-09-07

**Authors:** Yaw Amo Wiafe, Theophilus Kofi Adu-Bredu, Kwame Appiah-Denkyira, Charles Mawunyo Senaya

**Affiliations:** 1 Department of Obstetrics and Gynaecology, Komfo Anokye Teaching Hospital, Kumasi, Ghana,; 2 Department of Medical Diagnostics, College of Health Sciences, Kwame Nkrumah University of Science and Technology, Kumasi, Ghana

**Keywords:** Vasa previa, ultrasound, perinatal mortality, Africa

## Abstract

Vasa previa is characterised by unprotected umbilical vessels that are crossing the internal cervical os or lying close to it. When vasa previa is not detected during antenatal period, the perinatal outcome could be grievous. Ultrasound is the modality of choice in detection of vasa previa. Despite the increasing availability of ultrasound in modern times, its use in diagnosing vasa previa still remain very low in Africa. We present the sonographic findings and perinatal outcomes of three cases of vasa previa which were detected antenatally within a period of nine months in an African setting.

## Introduction

Vasa previa is characterised by unprotected umbilical vessels that crosses the internal cervical os or lying close to it. Because these vessels are not protected by Wharton´s jelly, they are susceptible to rupture and consequential fetal exsanguination leading to fetal or neonatal death. Another risk associated with vasa previa is the compression of fetal vessels by the presenting fetal part [1]. This may cause fetal hypoperfusion and acidaemia, with resultant asphyxia, stillbirth or neonatal death. Before ultrasound became widely available, vasa previa could rarely be diagnosed during antenatal period. The diagnosis was mostly made during labour and delivery when women with ruptured membranes present with painless vaginal bleeding and associated fetal distress [2]. Although other methods for detection exist, such as amnioscopy, Apt test and magnetic resonance imaging (MRI), ultrasound has become the preferred diagnostic tool [2] because it is non-invasive and accurate, and widely available. The first ultrasound diagnosis of vasa previa was reported over three decades ago. Despite the increasing availability of ultrasound in modern times, its use in diagnosing vasa previa still remain very low in Africa. Currently, existing knowledge about vasa previa is largely based on information from the western population. A systematic review conducted by Ruiter *et al*. found no reported use of ultrasound for the detection of vasa previa in sub-Saharan Africa [3]. However, the incidence of vasa previa is expected to increase in Africa as associated risk factors are increasing. We present the sonographic findings and perinatal outcomes of three cases of vasa previa which were detected antenatally within a span of nine months at our hospital.

## Patient and observation

**Figure 1:**
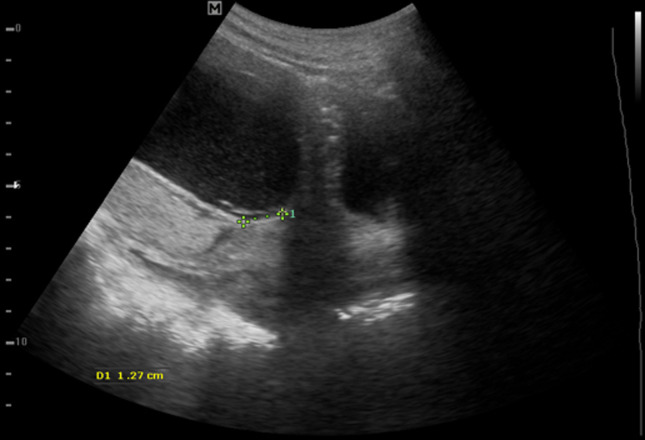
showing placenta about 1.3cm from the internal cervical os

**Figure 2:**
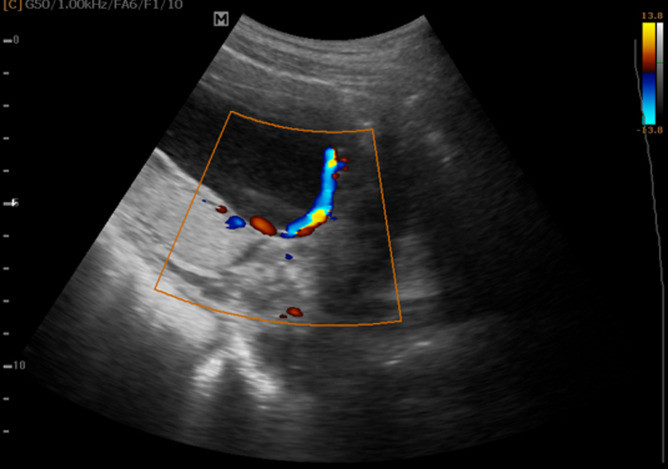
showing velamentous vessel overlying internal cervical os

**Case 1:** a 42-year-old Gravida 2 para 1 presented with intermittent painless bleeding per vaginam. At the time of admission, the gestation was 26 weeks + 3 days. Ultrasound examination revealed a single live intrauterine fetus. The placenta was located at the posterior uterine wall with the inferior edge seen about 1.3cm from the internal cervical os (Figure 1). A velamentous vessel was seen lying across the internal cervical os (Figure 2). Doppler analysis of the vessel showed arterial waveform. As the diagnosis was very clear on transabdominal approach, transvaginal ultrasound was not done. She was kept on admission till delivery. Dexamethasone was administered to the mother to stimulate fetal lung maturation. Follow-up transabdominal ultrasound showed that the vasa previa had persisted. A caesarean section was performed at 35weeks on account of heavy bleeding per vaginam. A healthy male baby was delivered with Apgar score of 7/10 at 1 minute and 8/10 at 5 minutes.

**Figure 3:**
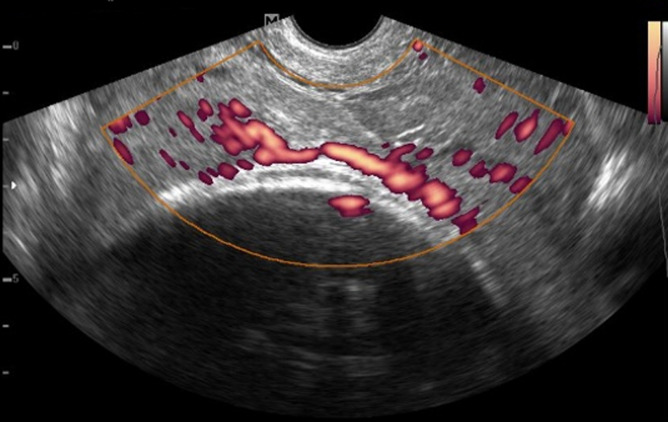
transvaginal ultrasound with power Doppler showing the fetal vessel between a bilobed placenta

**Figure 4:**
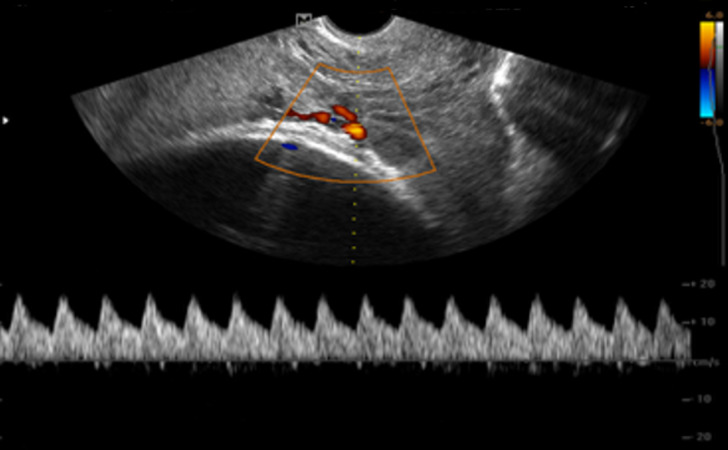
transvaginal ultrasound with pulse wave Doppler showing arterial waveform in fetal vessel

**Case 2:** a 35-year-old Gravida 5 para 3, plus 1 spontaneous miscarriage presented to the hospital for regular antenatal check-up. The gestational age was 37 weeks + 1day. She had no bleeding per vaginam, leaking amniotic fluid, or abdominal pain. Obstetric ultrasound was requested in planning towards delivery. A transabdominal ultrasound examination revealed a single live fetus with an estimated fetal weight of 3.5kg. While assessing the placenta, a succenturiate lobe was seen in the lower uterine segment. Evaluation with colour Doppler revealed a vessel overlying the internal cervical os. A further assessment with transvaginal ultrasound showed the vessel was between the succenturiate lobe and the main placenta on power Doppler (Figure 3). Additional evaluation with pulse wave Doppler revealed an arterial waveform which was consistent with a fetal arterial vessel (Figure 4). An elective caesarean section was performed the following day. A 3.5kg male baby was delivered. However, there was a poor Apgar score of 0/10 at one minute, necessitating neonatal resuscitation. Apgar score at 5 minutes was 1/10. The baby was intubated and admitted to the neonatal intensive care unit but died within 24 hours.

**Figure 5:**
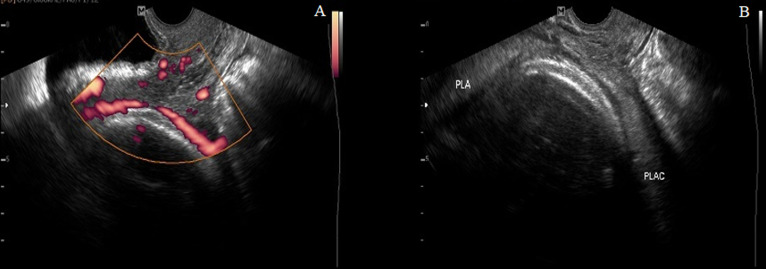
two images stacked side by side showing an interconnecting vessel overlying the internal cervical os on power Doppler (A) and low lying bilobed placenta (B)

**Figure 6:**
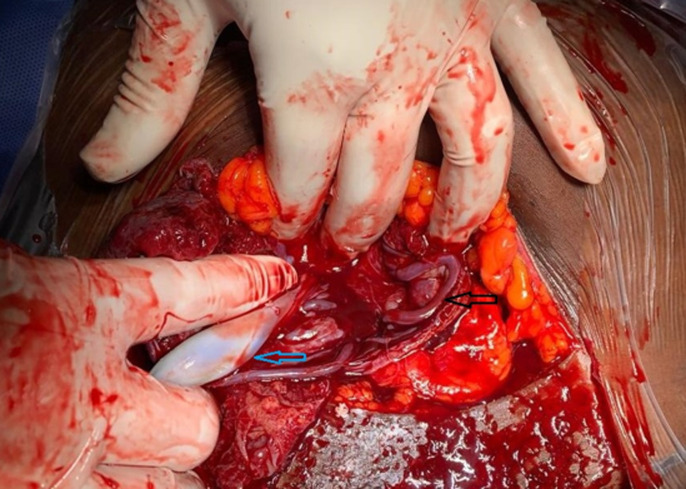
intraoperative image showing multiple aberrant umbilical vessels unprotected by Wharton´s jelly (black pointer) at the lower uterine segment: the blue pointer shows umbilical vessels protected by the Wharton´s jelly

**Case 3:** a 43-year-old Gravida 3 para 2, was referred to our tertiary hospital on account of advanced maternal age and low-lying placenta. The gestational age was 35 weeks + 3 days. An initial transabdominal ultrasound revealed a single live fetus with estimated weight of 2.9kg. A low lying bilobed placenta was seen at the anterior and posterior lower uterine walls without covering the internal cervical os. Further evaluation with transvaginal ultrasound revealed a vessel overlying the internal cervical os and connecting the two lobes of the placenta (Figure 5). Doppler assessment of the vessel demonstrated arterial flow. An elective caesarean section was performed three days later. A healthy female baby with a birthweight of 2.7kg was delivered. The Apgar scores were 8/10 at 1 minute and 9/10 at 5 minutes. Intraoperative findings revealed multiple umbilical vessels unprotected by the Wharton´s jelly on the fetal membrane (Figure 6).

## Discussion

Vasa previa is a relatively rare condition with a reported incidence of 0.6 per 1000 deliveries [4]. The rate is expected to increase because of increasing risk factors globally. Our records show an incidence of 3 in 5607 deliveries equivalent to 0.53 per 1000 deliveries which is similar to previously reported incidences. Vasa previa is associated with increased perinatal mortality. The mortality rates in pregnancies with undetected vasa previa is about 56%. However, when detected earlier during the antenatal period, the perinatal outcome improves to 97% [5]. Our perinatal outcome was 66.7% good with mortality rate of 33.3%. The outcomes were expected to be better because diagnoses were made antenatally and deliveries were done through caesarean section, although the affected case was diagnosed after 37 weeks. Notable among the risk factors are placenta previa or low-lying placenta, assisted reproduction, and twin gestation [2]. Placenta previa or low-lying placenta is strongly associated with vasa previa and had been reported to increase the risk by up to 78% [4]. All our cases had placenta previa or low-lying placenta. An important risk factor for placenta previa is advanced maternal age [6]. The prevalence of advanced maternal age in sub-Saharan Africa has been on the rise, making mothers more susceptible to placenta previa and hence vasa previa. In each of our cases, the maternal age was 35 years or older.

The advent of assisted reproduction which is now increasing in Africa [7], is also associated with increased risk of vasa previa. Rebarber *et al*. [8] reported a high incidence of 41% of vasa previa associated with assisted reproduction. Vasa previa has also been found to be associated with twin gestation. An incidence of 24% in twin gestation have been reported [9]. Africa has one of the highest twining rates in the world [9] and the advent of assisted reproduction is expected to increase the rate of twining. The combination of increasing advanced maternal age, the high incidence of twining in natural conception and the increasing use of assisted reproduction in Africa, may contribute to the expected increase in vasa previa in Africa. In assessing for vasa previa, the combination of transabdominal and transvaginal ultrasound improves detection rate [4]. In our first case, the diagnosis was made solely on transabdominal ultrasound. The transvaginal ultrasound was needed to confirm the diagnosis in the other two cases. Colour Doppler Imaging was found to be the most important application in both transabdominal and transvaginal diagnosis of vasa previa. Where colour Doppler imaging appears less sensitive, power Doppler helps to improve sensitivity as shown in our cases. The recommended management option of vasa previa is hospital admission by 30 - 32 weeks, antenatal steroids administration and delivery by elective caesarean section at 35 weeks before spontaneous rupture of membranes [10]. In two of our cases, diagnoses were made early and delivery was by caesarean section around 35weeks with good outcomes. In one case however, diagnosis was made at 37 weeks and elective section was done the next day. The outcome however was poor. The cause of the poor Apgar was not clear but could be due to compression of the unprotected vessels by the fetal head. We highly recommend targeted screening with ultrasound for vasa previa in all pregnant women with high risk factors, as early diagnosis and appropriate management will improve perinatal outcome.

## Conclusion

Vasa previa is associated with increased risk of perinatal mortality. Our records show an incidence of 0.53 per 1000 deliveries in our facility. This is comparable to previously reported incidences in the western world. The successful perinatal outcome of two out of three cases is associated with earlier diagnoses and delivery by caesarean section around 35 weeks. We highly recommend targeted screening with ultrasound for vasa previa in women with high risk factors in order to improve the perinatal outcome.
